# High-performance p-type V_2_O_3_ films by spray pyrolysis for transparent conducting oxide applications

**DOI:** 10.1038/s41598-024-52024-4

**Published:** 2024-01-22

**Authors:** Ardak Ainabayev, Brian Walls, Daragh Mullarkey, David Caffrey, Karsten Fleischer, Christopher M. Smith, Amy McGlinchey, Daniel Casey, Sarah J. McCormack, Igor Shvets

**Affiliations:** 1 School of Physics, Trinity College Dublin, College Green, Dublin 2, Dublin, D02 PN40 Ireland; 2 Centre for Research On Adaptive Nanostructures and Nanodevices, Trinity College Dublin, 43 Pearse St, Dublin 2, Dublin, D02 W085 Ireland; 3 Nazarbayev University, Qabanbay Batyr Ave 53, Astana, 010000 Kazakhstan; 4 Advanced Processing Technology Research Centre, Dublin City University, Glasnevin, Dublin, D09 K2WA Ireland; 5 Department of Civil, Structural and Environmental Engineering, School of Engineering, Trinity College Dublin, College Green, Dublin, D02 PN40 Ireland

**Keywords:** Applied physics, Chemical physics, Condensed-matter physics, Electronics, photonics and device physics, Electronic properties and materials, Electronic properties and materials, Structural properties, Synthesis and processing, Nanoscale materials, Materials for devices, Materials for energy and catalysis, Materials for optics

## Abstract

High-quality epitaxial p-type V_2_O_3_ thin films have been synthesized by spray pyrolysis. The films exhibited excellent electrical performance, with measurable mobility and high carrier concentration. The conductivity of the films varied between 115 and 1079 Scm^−1^ while the optical transparency of the films ranged from 32 to 65% in the visible region. The observed limitations in thinner films’ mobility were attributed to the nanosized granular structure and the presence of two preferred growth orientations. The 60 nm thick V_2_O_3_ film demonstrated a highly competitive transparency-conductivity figure of merit compared to the state-of-the-art.

## Introduction

P-type Transparent Conductive Oxides (TCOs) possess wide-ranging potential in various technological applications, including solar cells^1^, organic light-emitting diodes^2^, transparent thin-film transistors^3^, and more^4^. However, current p-type TCOs do not exhibit the necessary combination of electrical conductivity and transparency required for widespread industrial use, unlike their n-type counterparts^4–7^. To meet the demands of photovoltaics and water-splitting technologies, there is a crucial need for high-performance p-type TCOs capable of facilitating effective hole collection and enabling the fabrication of efficient transparent p-n junctions^8,9^. As a result, the development of a high figure of merit p-type TCO has become an urgent issue and a subject of intense research^4,6,10^.

**Figure 1:**
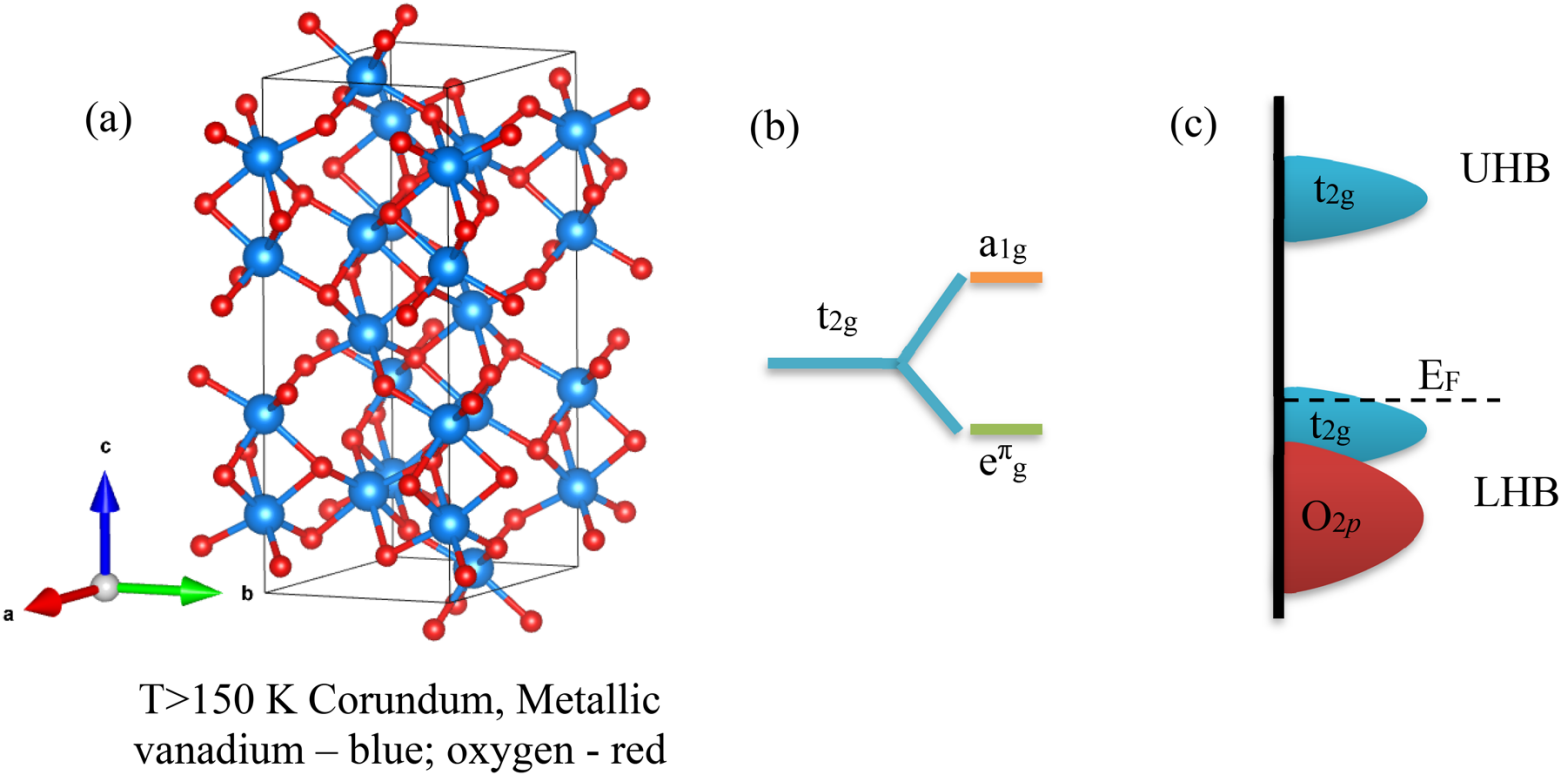
V_2_O_3_ crystal structure (**a**) above the MIT temperature (~ 150 K), (**b**) the simplified V_2_O_3_ *t*_*2g*_ band diagram and (**c**) a sketch of VB modification due to electron correlation in V_2_O_3_ (UHB, LHB are the upper and the lower Hubbard band, respectively).

In a recent study by Hu et al.^11^, it was demonstrated that pulsed laser deposited (PLD) grown V_2_O_3_ has the potential to serve as a new p-type TCO. V_2_O_3_ exhibits electron correlation due to the on-site Coulomb interaction, leading to changes in the valence band that promote p-type conductivity. Furthermore, the screened plasma energy in V_2_O_3_ is positioned below the visible region, resulting in enhanced optical transparency. At room temperature, V_2_O_3_ adopts a corundum structure with a space group of $R\overline{3 }c$ and lattice parameters of *a* = 4.942 Å and *c* = 13.99 Å^12^ (Fig. 1a). This material undergoes a Metal–Insulator Transition (MIT) at low temperatures (~ 150 K)^13^, where the corundum structure with metallic conductivity transforms into an insulating monoclinic structure with a space group of $C2/c$ ^14^. The lattice parameters in the monoclinic phase are *a* = 8.60 Å, *b* = 5.00 Å, *c* = 5.55 Å, and *β* = 123.1°. In V_2_O_3_, the *3d* bands split into twofold *e*_*g*_ and threefold *t*_*2g*_ bands. The *t*_*2g*_ bands further degenerate into low-lying doubly degenerate e^π^_g_ and nondegenerate a_1g_ bands due to the trigonal distortion of the rhombohedral lattice (Fig. 1b). In the metallic phase of V_2_O_3_, the valence band (VB) is composed of *e*^*π*^_*g*_ and *a*_*1g*_ bands, and it is partially occupied by two *t*_*2g*_ electrons (Fig. 1c). The Fermi level is within the upper VB, explaining the p-type conduction of V_2_O_3_ with high conductivity (*σ*) and hole concentration (*n*_*h*_). Unlike most p-type TCOs, this characteristic of V_2_O_3_ allows for circumventing issues associated with hole doping, such as the high formation energy of native acceptors and self-compensation effects^10,11,15^.

The renormalization factor Z_*k*_, defined as the ratio of the free non-interacting electron effective mass (m_*band*_) to the renormalized electron effective mass (m^∗^) that incorporates electron–electron interaction, can be used to quantify the strength of electron correlation. In the case of V_2_O_3_, Z_*k*_ is equal to 0.12^11^. The hole effective mass in V_2_O_3_ is larger than that in conventional TCO materials and can be estimated as m* = m_*band*_/Z_*k*_ ≈ 8.3m_*band*_. This increase of the effective mass occurs due to the localization of vanadium 3*d* electron orbitals near the VB maximum. Although a large hole effective mass typically hampers hole mobility, the high hole concentration (n_*h*_) compensates for it, resulting in high conductivity for V_2_O_3_^10,11,15^. To achieve optical transparency in the visible range, the reflection edge in the material should be below 1.75 eV, while the strong interband optical transition should be above 3.25 eV. The screened plasma energy *ħω*_*p*_, which describes the free carrier reflection edge, can be calculated as *ħω*_*p*_ = (*e*^2^/*ε*_0_*ε*_*r*_)^1/2^(n*/*m^∗^)^1/2^, where n is the carrier concentration, *e* is the elemental charge, *ε*_0_ is the vacuum permittivity, and *ε*_*r*_ is the relative permittivity. The enhanced m^*^ = 8.3m_*band*_ in V_2_O_3_, resulting from electron correlation, tunes the n/m^∗^ ratio in such a way that *ħω*_*p*_ shifts below 1.75 eV, enabling optical transparency of V_2_O_3_ in the visible range. Additionally, the optical absorption in the visible region of V_2_O_3_ is governed by the *d-d* transition from occupied t_*2g*_ to empty t_*2g*_ bands, which are low, allowing V_2_O_3_ films to remain sufficiently transparent. The upper limit of transparency (E_*H*_), where strong absorption is observed at a photon energy around 4 eV, is controlled by charge transfer excitations from O_*2p*_ to t_*2g*_ bands in the ultraviolet region^10,11,15^.

Limited research has been conducted on the deposition of p-type V_2_O_3_ utilizing simpler growth techniques than various physical vapour deposition (PVD) methods. Consequently, there is a need to explore alternative fabrication approaches offering the synthesis of the high-quality p-type V_2_O_3_. In this study, we present, to our knowledge, the successful synthesis of high-quality p-type V_2_O_3_ TCO thin films on *c*-plane Al_2_O_3_ substrate using a single-step spray pyrolysis (SP) method. The structural, optical, and electrical properties of V_2_O_3_ of various thicknesses were characterized in relation to TCO application. The deposited p-type V_2_O_3_ films exhibited an epitaxial structure with the substrate and a conductivity of 1079 Scm^−1^ (0.05 M, 60 nm thick sample), which is the highest reported for a solution-based thin film growth method to date, without requiring post-deposition treatment. Furthermore, the grown V_2_O_3_ samples demonstrated quantifiable mobility through simple dc Hall measurements and exhibited relative atmospheric stability. Although the Figure of Merit (FoM) by Gordon and Haacke of the SP-grown V_2_O_3_ thin film (0.05 M, 60 nm thick sample) was smaller compared to their PLD-grown counterparts^11^, the obtained FoM is the highest reported for SP-grown p-type TCOs and surpasses several other PVD-grown p-type TCOs. We attribute the primary limiting factor in electrical performance to film morphology and growth orientation, which can be linked to inherent factors in our deposition setup. Nevertheless, the low-cost, non-vacuum, and high-yield spray pyrolysis method^16,17^ stands out as a simple, scalable, and cost-effective approach for fabricating p-type V_2_O_3_, which is important considering the potential applicability of p-type V_2_O_3_ in device applications such as rectifiers, photodetectors, and solar cells.

## Methods

The details of the chamber geometry, nozzle type, and other parameters, experimental details of this study are provided elsewhere^18^. In summary, all thin films were deposited on single-side polished *c*-Al_2_O_3_ substrates (10 × 10 mm, 1 mm thick). The precursor used was vanadium (III) acetylacetonate V(C_5_H_7_O_2_)_3_ (Sigma Aldrich), which was dissolved in methanol at varying (0.05, 0.025, 0.015, 0.10, and 0.005 M) molarities (concentrations) to achieve different film thicknesses. The thickness of the sample grown with a solution molarity of 0.05 M was determined to be 60 nm using ellipsometry, which will be discussed in detail later. Under the assumption that the grown film thickness is proportional to molarity^19^, the thicknesses of the remaining samples are estimated to be: 0.025 M—30 nm; 0.015 M—20 nm; 0.01 M—15 nm; 0.005 M—10 nm. To ensure clarity throughout the text, the thicknesses will be explicitly referenced and utilized. It should be noted that vanadium (III) acetylacetonate exhibits high solubility in methanol without any observable precipitation. The solution was sprayed onto a heated substrate at a heater temperature of 718 ± 5 K using compressed nitrogen gas with a flow rate of 15 L/min. The heater temperature was controlled using a PID controller, and a type K thermocouple attached to the heater surface provided temperature feedback.

To analyze the out-of-plane orientations of the films, as well as the in-plane orientation for determining the epitaxial relationship between the film and substrate, symmetric θ/2θ-scans and φ-scans were performed using a Bruker D8 Advance X-ray diffraction (XRD) system with a Cu Kα source. The XRD system was equipped with a Ge (002) double bounce monochromator. Additionally, Raman measurements were conducted at room temperature using a Witec Alpha 300R instrument with an excitation wavelength of 532 nm and a power level below 1 mW to prevent local heating effects. Surface morphology and roughness were investigated using Bruker atomic force microscopy (AFM) in tapping mode with a probe tip diameter of 20 nm. Optical properties were characterized using UV–VIS spectrometer (PerkinElmer 650S) and ellipsometry (SOPRA GESP5). For electrical characterization at room and low temperatures, a custom-built four-point probe station with a square Van-der-Pauw geometry and silver paint-based contacts was used. The dc Hall measurements were performed with a maximum magnetic field of 800 mT. All electrical measurements were carried out using a Keithley 2400 source meter.

## Results and discussion

**Figure 2:**
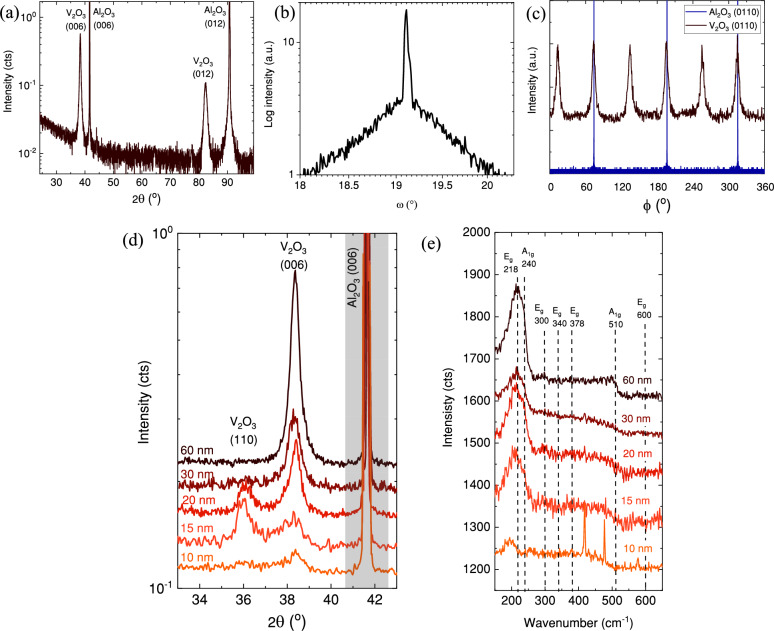
(**a**) Full range XRD θ/2θ scan of 60 nm (0.05 M) V_2_O_3_ (0001) thin film. (**b**) XRD rocking curve measurement for the 60 nm (0.05 M) V_2_O_3_ (0001) thin film. (**c**) XRD φ-scan measurements for the 60 nm (0.05 M) V_2_O_3_ (0110) planes and Al_2_O_3_ (0110) planes. Narrow (**d**) XRD and (**e**) Raman scans with data for all samples have been stacked according to the thickness. Shaded area denotes *c*-Al_2_O_3_ (0006) XRD peak.

**Figure 3:**
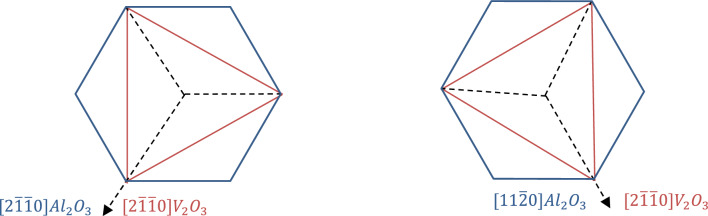
Schematic showing the alignment of two different domains of V_2_O_3_ on the Al_2_O_3_.

Figure 2a shows the out-of-plane XRD pattern of the 60 nm (0.05 M) V_2_O_3_ sample grown on *c*-Al_2_O_3_ substrate. For this sample, only (000n) order peaks for the V_2_O_3_ thin film and Al_2_O_3_ substrate are observed, confirming a single crystalline phase and *c*-axis oriented growth of V_2_O_3_. The high quality of the 60 nm (0.05 M) V_2_O_3_ sample is further validated through rocking curve measurements (ω scan), revealing a full width at half maximum around 0.05° (Fig. 1b). To determine the epitaxial relationship between film and substrate, φ scans of the V_2_O_3_ film (0110) reflection and of the substrate (0110) reflection were performed (Fig. 2c). Since Al_2_O_3_ is rhombohedral, the (0110) reflection shows three-fold symmetry. The presence of six peaks for the (0110) reflection of V_2_O_3_ instead of expected three implies the presence of the two domains of V_2_O_3_. The equal intensity of each of the six observed peaks indicates that each of the two domains are present in approximately equal quantities. The schematics of the two V_2_O_3_ domain arrangements on *c*-Al_2_O_3_ is shown in Fig. 3. One of the V_2_O_3_ domain’s basal plane is aligned with that of *c*-Al_2_O_3_, whereas for the other domain there is (60°) 180° in-plane rotation. The following epitaxial relation was derived: V_2_O_3_ $(0\; 0\; 1)$ ‖ Al_2_O_3_ $(0\; 0\; 1)$; V_2_O_3_ $[2\;\overline{1 }\;\overline{1 }\;0]$ ‖ Al_2_O_3_ $[2\;\overline{1 }\;\overline{1 }\;0]$ or V_2_O_3_ $[2\;\overline{1 }\;\overline{1 }\;0]$ ‖ Al_2_O_3_ $[1\;1\;\overline{2 }\;0]$.

However, altering the molarity, and consequently, the thickness, of the samples leads to changes in the observed reflexes of the V_2_O_3_ thin films (Fig. 2d). When the thickness is less than 30 nm (0.025 M) the (110) reflection is present in addition to the (0006) reflection. The intensity of the XRD reflections reduces gradually with decreasing thickness because the scattered intensity is proportional to the sample thickness. The peak broadening without a shift in position is also observed as thickness is reduced, due to a thickness decrease from 60 to 5 nm. We propose that an increased thickness leads to the formation of an epitaxial thin film. However, the appearance of an additional peak to (001), such as the (110) below a thickness of 30 nm, suggests a deviation from epitaxial growth at smaller thicknesses. To the best of our knowledge, the occurrence of V_2_O_3_ (110) orientation on *c*-Al_2_O_3_ has not been observed through physical vapor deposition (PVD) methods^20,21^. Nevertheless, in a study^22^ it was reported the presence of the (110) peak in the XRD pattern of V_2_O_3_ grown using a sol–gel method on *c*-Al_2_O_3_. It seems that, particularly in V_2_O_3_ prepared from a solution, there is a competition in preferential growth orientations between (110) and (001) planes. In our experiments involving samples of varying thickness, a consistent trend emerged. Larger thicknesses seemed to promote the exclusive growth of the (001) orientation, whereas thinner films exhibited the appearance of the (110) peak in addition to the (006) peak. Understanding such changes requires additional measurements, as they could occur due to various factors, such as strain relaxation or surface energy minimization. Typically the thin film texture for SP grown films strongly depends on substrate, solvent and precursor choice^18^, but in the case of strongly textured growth an influence on growth rate (affected by molarity) is also frequently observed^23^. The biaxial in-plane strain values for (110) on *c*-Al_2_O_3_ are 4.1% and 1.94%, whereas for (001), they are both 4.1%. In this context, there is a comparable likelihood of initiating either (110) or (001) growth orientations. The growth rate is a key factor influencing the dynamics of surface diffusion, which involves the movement of adatoms across the substrate surface. Specifically, for (110) growth orientation, adatoms need to traverse a larger distance across the substrate surface. There is a possibility that a slower growth rate might promote (110) growth orientation because adatoms would have ample time for lateral diffusion before being incorporated into the film. Conversely, higher growth rates could limit the time available for lateral diffusion, resulting in the incorporation of adatoms into the film and promoting (001) orientation. Deposition techniques that introduce a higher flux of adatoms to the substrate surface, such as sputtering or molecular beam epitaxy, generally yield higher growth rates compared to other methods, thereby probably favouring the growth of (001) orientation. For solution-based thin film growth techniques like spray pyrolysis, the growth rate is partially controlled by the concentration of the precursor in the solvent. Lower concentrations encourage a slower growth rate, enabling the fabrication of thinner films with the presence of (110) growth orientation. The question of why (110) growth orientation persists to substantial thicknesses in V_2_O_3_ obtained by a solution-based process is one that we plan to address in future research.

Additionally, Raman spectroscopy was performed to confirm the phase identification of the V_2_O_3_ samples (Fig. 2e). V_2_O_3_ has 7 Raman-active modes (2A_1g_ + 5E_g_) for a crystal with a corundum structure^24^. First A_1g_ mode is experimentally observed at 240 cm^−1^ and it is related to the vibration of the vanadium atoms along the z-axis towards the oxygen triangle plane and back. While the second A_1g_ mode observed at 510 cm^−1^ belongs to the vibration of the oxygen atoms towards the z-axis and back in the basal plane. The first E_g_ mode at 218 cm^−1^ is attributed to the out-of-phase movement of vanadium in the basal plane. In phase motion of vanadium atoms yield the second E_g_ mode at 300 cm^−1^. The remaining E_g_ modes describe the complex motion of oxygen atoms and are found at 340 and 600 cm^−1^. Another E_g_ mode at 378 cm^−1^ was not observed experimentally but it was theoretically predicted^24^. In our Raman measurements, broad an asymmetric peak covering a range of 160–250 cm^−1^ is clearly observable, probably due to convolution of the first E_g_ (218 cm^−1^) and A_1g_ (240 cm^−1^) modes. This broad peak is redshifted as thickness decreases, possibly due to strain^15^. The remaining expected peaks are not that pronounced in intensity or absent, which was observed for V_2_O_3_ thin film in another study^15^. The Raman peak around 418 and 479 cm^−1^, related to the *c*-Al_2_O_3_ is absent for all films except the thinnest (10 nm), generally indicating that the coverage of the thin films is continuous and dense.

**Figure 4:**
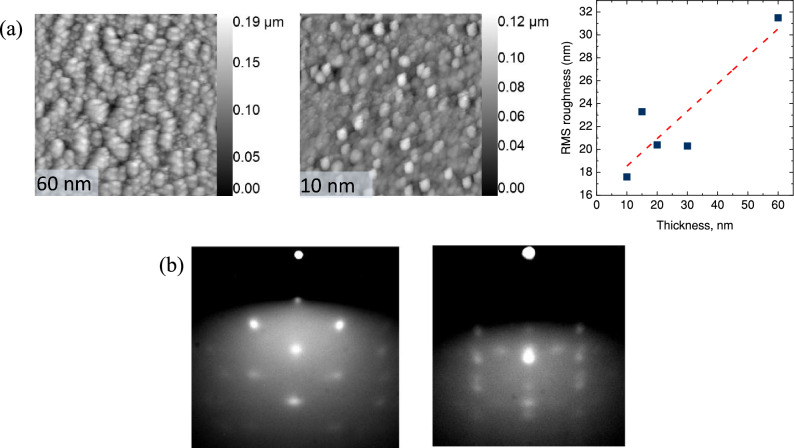
(**a**) 5 × 5 µm area AFM images of 60 and 10 nm thick SP-grown V_2_O_3_ samples and AFM measured RMS roughness of the V_2_O_3_ thin films. (**b**) RHEED patterns of the SP-grown V_2_O_3_ 60 nm sample (along Al_2_O_3_ $[1\;1\;\overline{ 2 }\;0]$ left and Al_2_O_3_ $[1\;0\;\overline{1 }\;0]$ right).

The AFM scans reveal that SP-grown V_2_O_3_ films exhibit a nanosized granular structure. Comparing the roughness of the samples, it is evident that the roughness increases as the thickness increases (Fig. 4a). However, attempts to obtain an x-ray reflectivity oscillations signal from any sample for thickness determination were unsuccessful, presumably due to the high surface roughness.

Figure 4b shows RHEED images taken along the Al_2_O_3_ $[1\;1\;\overline{2 }\;0]$ and Al_2_O_3_ $[1\;0\;\overline{1 }\;0]$ high symmetry directions of a 60 nm thick V_2_O_3_ sample. RHEED patterns were obtained post-deposition in a UHV chamber with a pressure less than 5 × 10^−9^ mbar, using an electron beam energy and current of 20 keV and 1.5 A, respectively. The pattern shown in Fig. 4b displays something between a transmission spot pattern and that of modulated streaks, which is characteristic of a film surface consisting of continuous 3-D islands with a rough surface. This correlates well with the roughness and granular morphology of this sample determined via AFM.

**Figure 5:**
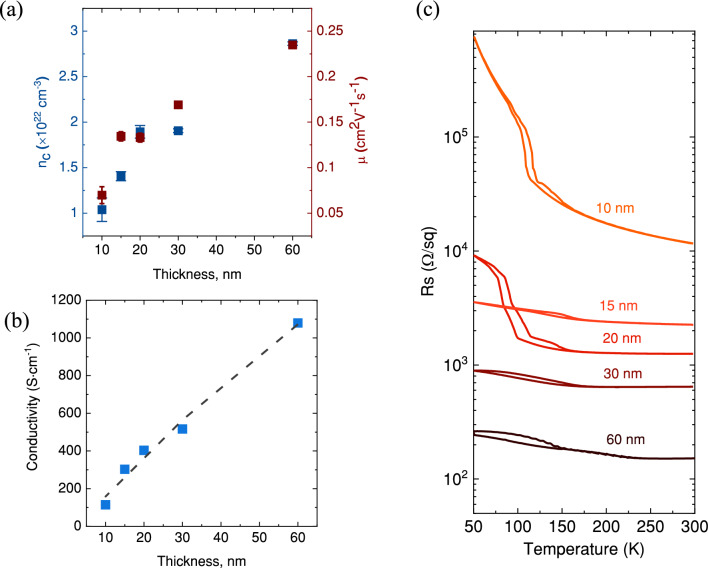
Room temperature (**a**) hole concentration, mobility, and (**b**) electrical conductivity. (**c**) Temperature-dependent sheet resistance.

Figure 5a, b and c demonstrate that hole concentration, mobility, conductivity and sheet resistance exhibits a strong dependence on the thickness (molarity of the solution). Consistent with previous studies^11,15^, the SP-grown V_2_O_3_ films exhibit p-type conductivity with high hole concentration (1.04 × 10^22^–2.86 × 10^22^ cm^3^) and low mobility (0.07–0.24 cm^2^/Vs) values. In comparison to PLD-grown p-V_2_O_3_^11^, SP grown samples demonstrate somewhat lower values of the hole concentration and mobility, resulting in lower conductivity values ranging from 115 to 1079 Scm^−1^. Notably, the sample with the largest thickness exhibits a hole concentration of 2.86 × 10^22^ cm^3^ and mobility of 0.24 cm^2^/Vs, values which are similar to the hole concentration (5.28 × 10^22^ cm^3^) and mobility (0.24 cm^2^/Vs) of the PLD-grown sample^11^. However, as thickness decreases the hole concentration remains in the same order of magnitude as PLD grown samples^11^, but mobility decreases more rapidly. In general, mobility reduces with decrease in thickness because of the overall increase in scattering centres. In addition to the size effect, the possible reason for more rapid mobility reduction for SP grown V_2_O_3_ can be nanosized granular morphology and the appearance of two growth orientations for samples with smaller thicknesses. This is in contrast to the PLD-grown V_2_O_3_ samples^11^, which exhibit a single orientation and very smooth, non-granular morphology across all thicknesses.

Mobility in a single crystal depends on the orientation; in the case of a polycrystalline sample, it can be affected by grain boundaries between grains at different orientations and texture. In this regard, the presence of (110) growth orientation in addition to (001) growth orientation might adversely affect mobility in case of SP-grown V_2_O_3_. Furthermore, the nanosized granular morphology serves as an effective scattering source for hole transport, thereby reducing mobility. This phenomenon was also observed in a previous study^17^ conducted by the authors, which investigated zinc tin oxide TCO films deposited using the same SP equipment. In that study^17^, the limited mobility of charge carriers in zinc tin oxide was attributed to the granular nature of the films.

V_2_O_3_ is known to undergo a MIT at 150 K. Therefore, low-temperature resistance measurements were conducted for all samples to investigate this behaviour (Fig. 5c). It is observed that all samples exhibit a transition in resistivity, shifting towards a more insulating state at reduced temperature, with a characteristic hysteresis loop indicative of a first-order phase transition at approximately 150 K. It should be noted that the observed MIT in all samples is suppressed, and the magnitude of the transition and its width are smaller than single crystal bulk V_2_O_3_. The thinner films, demonstrate a more pronounced MIT compared to the thicker samples. However, the 15 nm thick sample deviates from this pattern. Evidently, this particular sample exhibits a distinct prevalence of (110) crystallographic orientation in comparison to the other samples, which potentially underlies this unique behavior^25^. It should be noted that a growth orientation of (110) was achieved on the *a*-Al_2_O_3_ substrate in the studies^20,21^ using PVD techniques, resulting in a pronounced MIT at approximately 150 K. In contrast, our samples deviate from this approach as we employ a solution spraying method, resulting in non-epitaxial V_2_O_3_ films with a thickness of 15 nm on *c*-Al_2_O_3_ with two growth orientations, (110) and (001). This difference complicates direct comparisons with the epitaxial films discussed in the aforementioned studies^20,21^. The presence of internal strain and stress, stemming from crystal orientation mismatches in materials with several growth orientations or texture, has the potential to alter the structure and MIT properties of V_2_O_3_^26–28^. Shifts in the MIT temperature and stabilization of the metallic phase in thicker films have been observed in PLD-deposited V_2_O_3_ films^29–31^. Allimi et al^29^ suggested that the observed behavior in the thicker films might be related to specific defect microstructures that induce localized conditions analogous to the application of a hydrostatic pressure in bulk. In order to comprehensively elucidate the intricate relationship between resistance variation and temperature in V_2_O_3_, further microstructural analysis is required^29–31^.

**Figure 6:**
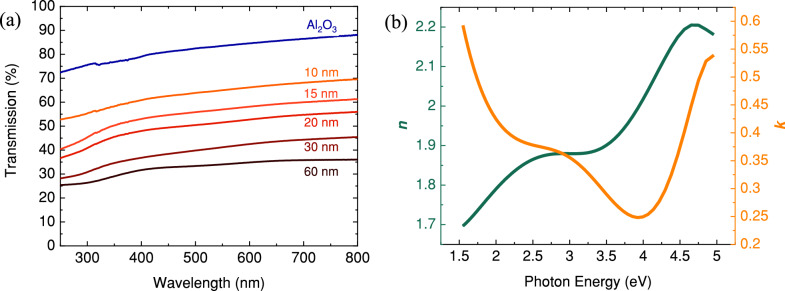
(**a**) UV–Vis transmittance spectra for the Al_2_O_3_ substrate and V_2_O_3_ thin films with different thicknesses and (**b**) 60 nm thick (0.05 M) V_2_O_3_ film’s refractive index *n*, and extinction coefficient *k*, as a function of photon energy in the wide spectral range modelled from the ellipsometry data. The larger step in transmission reduction from the bare Al_2_O_3_ to 10 nm V_2_O_3_ is caused by the larger refractive index of V_2_O_3_ compared to Al_2_O_3_ and hence an increased reflection loss.

Given the potential use of the films as TCO their optical properties are also of importance. Figure 6a summarises the transmission measurements for the spray grown V_2_O_3_ samples. Room temperature transmission ranges 32–65% for the visible and near-infrared ranges. The transparency of the V_2_O_3_ thin films is reduced with increasing film thickness due to the unavoidable optical absorption in the visible region. The behaviour of light passing through a thin film can be understood by considering its complex refractive index^32^. This index, denoted by *ñ*, consists of two parts: the real refractive index (*n*) and the imaginary extinction coefficient (*κ*). To determine the thickness of the sample grown with a solution molarity of 0.05 M and its complex refractive index, room temperature ellipsometry measurements were performed. The thickness of the sample grown with a solution molarity of 0.05 M was determined to be 60 nm.

The transmission of light through a sample depends on its reflectivity and absorption coefficient. Figure 6b illustrates the variation of *n* and *κ* for the 60 nm V_2_O_3_ thin film as a function of photon energy. The extinction coefficient *κ* is directly related to the absorption coefficient *α* of the material, where *α* = 4*πκ*/*λ* and *λ* is the wavelength of light in vacuum^32^. A lower value of *κ* corresponds to lower *α*, indicating higher transparency. In the visible range (around 2.0 eV), V_2_O_3_ exhibits a *κ* value of approximately 0.4, suggesting good transparency.

However, absorption increases at photon energies above 4.5 eV and in the infrared range below 2 eV due to interband transitions and free carrier excitation, respectively. Determining the bandgap of V_2_O_3_ through the absorption coefficient we consider unreliable due to the complex interband states^33^ in its band structure. Although V_2_O_3_ possesses interband states and behaves as a metal at room temperature, it exhibits low absorption below 4.5 eV because of electron correlation effects, which can shift the plasma frequency below 1.75 eV, resulting in optical transparency in the visible range^11^. Despite being highly conductive, V_2_O_3_ thin films have low refractive index values in the infrared and visible spectral regions. This property makes them potentially suitable as buffer layers in organic light-emitting diodes or solar cell devices where a reduced refractive index or lower work function is required for the transparent contact^34^. For instance, V_2_O_3_'s refractive index of 1.8 in the visible region is ideal for minimizing reflective losses at glass/TCO interfaces while maintaining conductivity^35^.

In crystalline materials, the electrical properties can vary significantly between thinner films and films with micrometer thickness, depending on the growth method and conditions. As a result, deriving a representative FoM for the material can be challenging, and it becomes harder to differentiate between systematic issues in the FoM calculation and genuine material changes. This complexity arises due to the differences in grain sizes and associated electrical properties between the two film thickness regimes.

The preferable way of evaluating TCO performance is based on calculation of the FoM by using its conductivity and absorption coefficient FoM_TR_ = σ/α = −1/(R_s_ ln(T + R))^46^. For the sample V_2_O_3_ with 60 nm thickness this FoM will be 2776.7 µS. This expression provides a measure of the TCO's quality, independent of its thickness, and has units of Siemens. It can be determined by measuring the thin film's sheet resistance (R_s_), transmittance (T), and reflectance (R) without requiring detailed knowledge of the film thickness. This makes it an ideal quantity for experimental screening purposes. In epitaxial systems where the TCO's microstructure remains unaffected by the sample thickness, FoM_TR_ serves as a reliable parameter for comparing the performance of p-type TCOs. When using this FoM the challenge arises when UV–VIS spectrometers lacking the capability to perform reflectance measurements is utilized, in this case the FoM is reduced to the −1/R_s_ ln(T). FoM loses its thickness independence, making it difficult to compare samples with different thicknesses reliably. Moreover, relying solely on transmission data can be problematic since it becomes challenging to distinguish between unwanted absorption losses and acceptable reflection losses. Another concern is that the FoM alone does not provide information about a material's band gap or hole mobility, which are crucial for optoelectronic devices. Therefore, a high FoM does not necessarily translate to enhanced efficacy in a specific device^46^. To evaluate the performance of SP-grown V_2_O_3_ as a p-type TCO, two commonly used FoM were calculated: the Haacke^47^ FoM (FoM_H_ = T^10^/R_s_) and the Gordon^48^ FoM (FoM_G_ = −1/(R_s_ ln T)). The FoM calculations were performed for the sample grown from a 0.05 M solution, which had its thickness (60 nm thick) precisely determined through ellipsometry measurements (in contrast to the estimated thickness of the other samples). Table 1 presents a comparison of the FoM values for various p-type materials, including the 0.05 M 60 nm thick V_2_O_3_ sample. Although our SP-grown V_2_O_3_ exhibits a smaller FoM compared to the remarkably high FoM of PLD-grown V_2_O_3_ samples^11,15^, it still stands out as being comparable to or even higher than other p-type TCOs obtained through both chemical and physical deposition methods. This demonstrates the capability of the spray pyrolysis technique to produce high-performance p-type TCOs, particularly electron-correlated materials like V_2_O_3_. We propose that further improvements in SP-grown V_2_O_3_ as a p-type TCO can be achieved by better control over the growth conditions. It is crucial to prevent the presence of multiple growth orientations and strive for a smooth surface with larger grains, as these factors can significantly enhance mobility, hole carrier concentration, and ultimately, conductivity.

**Table 1:** List of the performance of reported best p-type TCOs in the literature and the 60 nm thick (0.05 M) V_2_O_3_ thin film in this work. (t is the film thickness; R_s_ is the sheet resistance; T is the transmittance in visible range; σ is the electrical conductivity.)

Materials	t, (nm)	T_av_, (%)	R_s_, (kΩ/sq)	σ, (Scm^−1^)	FOM_G_ (MΩ^−1^)	FOM_H_ (MΩ^−1^)	Deposition
CuCrO_2_^36^	140	50	–	17	2300 mS (FoM = $\sigma /\alpha$)	–	PI-MOCVD
Cu_0.66_Cr_1.33_O_2_^37^	200	60	–	100	–	–	DLI-MOCVD
Cu_*x*_CrO_*y*_^38^	90	55	–	12	350 μS (FoM = $\sigma /\alpha$)	0.15	Spray pyrolysis
Mg:CuCrO_2_^39^	100	63	71	1.4	–	0.15	RF-sputtering
CuCr_0.95_Mg_0.05_O_2_^40^	250	30	0.182	220	4564	0.03	RF-sputtering
CuCrO_2_^41^	124	27	–	103	2040 µS (FoM = $\alpha /\sigma$)	0.28	Spray pyrolysis
V_2_O_3_^11^	56	40	0.084	2122	12,992	1.25	PLD
V_2_O_3_^15^	40	34.6	0.042	5896	22,222	0.58	PLD
La_2/3_Sr_1/3_VO_3_^42^	24	61.1	0.51	816.3	3977	14.21	PLD
Li_x_NbO_2_^43^	81	38	1.07	115	962	0.085	PLD
Ca_3_Co_4_O_9_^44^	50	50	1.46	136.9	988	0.669	Solid-state reaction
Bi_2_Sr_2_CaCu_2_O_y_^45^	30	57	0.8	427	2279	4.64	Spin-coating
This work	60	32	0.154	1079	5699	0.073	Spray pyrolysis

## Conclusions

In summary, we demonstrated successful growth of epitaxial p-type V_2_O_3_ thin films exhibiting excellent electrical performance for films deposited by inexpensive chemical synthesis methods. The highest-performing sample achieved a conductivity of 1079 Scm^−1^ (0.05 M, 60 nm thick V_2_O_3_ sample) obtained in single step without any post-deposition treatment is the highest reported for a solution-based thin film growth method to date. The deposited p-type V_2_O_3_ films have measurable mobility (0.07–0.24 cm^2^/Vs) and high carrier concentration (1.04 × 10^22^–2.86 × 10^22^ cm^3^) with a conductivity that varies between 115–1079 Scm^−1^. The optical transparency is in the range of 32–65%. The transparency is on the lower side at larger thicknesses and it is improving at small thicknesses. The FoM value of the V_2_O_3_ film (0.05 M, 60 nm thick sample) is found to be highly competitive even with several other PVD deposited p-type TCOs despite the constraints arising from the specifics of the utilized deposition system. We suggest that nanosized granular structure along with the presence of more than one preferred growth orientation in particular limits mobility. The more specialized system with a superior nebulizer could help overcome these limitations to push the conductivity and transparency of the SP grown V_2_O_3_ towards higher values. In general, the high conductivity and acceptable transparency at low thicknesses make V_2_O_3_ quite suitable as an active element facilitating the transport of photogenerated holes in solar cells. Future research should explore the deposition of V_2_O_3_ films on non-single crystal and inexpensive substrate such as glass while maintaining similar electrical and optical performance. Furthermore, exploring the physical mechanism responsible for the emergence of (110) growth orientations alongside (001) growth orientation in the grown V_2_O_3_ samples presents a very interesting question for future works.

## Data Availability

The data that support the findings of this study are available from the corresponding author upon reasonable request.
